# DNA Methylation Biomarkers as Prediction Tools for Therapeutic Response and Prognosis in Intermediate-Stage Hepatocellular Carcinoma

**DOI:** 10.3390/cancers15184465

**Published:** 2023-09-07

**Authors:** Chang-Yi Lu, Chih-Yang Hsiao, Pey-Jey Peng, Shao-Chang Huang, Meng-Rong Chuang, Hung-Ju Su, Kai-Wen Huang

**Affiliations:** 1 Phalanx Biotech Group, Hsinchu 300, Taiwan; cy.lu@neocore.com.tw (C.-Y.L.); sc.huang@neocore.com.tw (S.-C.H.); richardsu@phalanxbiotech.com (H.-J.S.); 2 Department of Surgery and Hepatitis Research Center, National Taiwan University Hospital, Taipei 100, Taiwan; 3 Graduate Institute of Clinical Medicine, College of Medicine, National Taiwan University, Taipei 100, Taiwan; 4 Department of Traumatology, National Taiwan University Hospital, Taipei 100, Taiwan; 5 Center for Functional Image and Interventional Therapy, National Taiwan University, Taipei 100, Taiwan

**Keywords:** DNA methylation, hepatocellular carcinoma, locoregional therapy, tumor biomarker

## Abstract

**Simple Summary:**

Patients with hepatocellular carcinoma are often monitored using serum markers such as alfa-fetoprotein. However, serum markers are not always accurate in predicting treatment success or the future course of the disease. In this research, we looked at the potential of DNA methylation as a new way to gauge treatment outcomes in patients with intermediate-stage hepatocellular carcinoma. We found that using these DNA changes might be more precise than just relying on serum markers. Even better, when we combined the information from both DNA methylation and serum markers, our predictions improved further. These discoveries could help doctors better personalize treatment plans, especially for patients who need close monitoring or additional therapy after their initial treatment.

**Abstract:**

Introduction: Alfa-fetoprotein (AFP), as the main serum tumor marker of hepatocellular carcinoma (HCC), is limited in terms of specificity and ability to predict outcomes. This study investigated the clinical utility of DNA methylation biomarkers to predict therapeutic responses and prognosis in intermediate-stage HCC. Methods: This study enrolled 72 patients with intermediate-stage HCC who underwent locoregional therapy (LRT) between 2020 and 2021. The immediate therapeutic response and disease status during a two-year follow-up were recorded. Analysis was performed on 10 selected DNA methylation biomarkers via pyrosequencing analysis of plasma collected before and after LRT. Results: Analysis was performed on 53 patients with complete responses and 19 patients with disease progression after LRT. The mean follow-up duration was 2.4 ± 0.6 years. A methylation prediction model for tumor response (MMTR) and a methylation prediction model for early progression (MMEP) were constructed. The area under the curve (AUC) for sensitivity and specificity of MMTR was 0.79 for complete response and 0.759 for overall survival. The corresponding AUCs for sensitivity and specificity of AFP and protein induced by vitamin K absence-II (PIVKA-II) were 0.717 and 0.708, respectively. Note that the MMTR index was the only significant predictor in multivariate analysis. The AUC for sensitivity and specificity of the MMEP in predicting early progression was 0.79. The corresponding AUCs for sensitivity and specificity of AFP and PIVKA-II were 0.758 and 0.714, respectively. Multivariate analysis revealed that platelet count, beyond up-to-7 criteria, and the MMEP index were strongly correlated with early tumor progression. Combining the indexes and serum markers further improved the predictive accuracy (AUC = 0.922). Multivariate analysis revealed the MMEP index was the only independent risk factor for overall survival. Discussion/Conclusions: This study indicates that these methylation markers could potentially outperform current serum markers in terms of accuracy and reliability in assessing treatment response and predicting outcomes. Combining methylation markers and serum markers further improved predictive accuracy, indicating that a multi-marker approach may be more effective in clinical practice. These findings suggest that DNA methylation biomarkers may be a useful tool for managing intermediate-stage HCC patients and guiding personalized treatment, particularly for those who are at high risk for close surveillance or adjuvant treatment after LRT.

## 1. Introduction

The worldwide incidence of hepatocellular carcinoma (HCC) is increasing, and the mortality rate is high [1]. Treatment choice and prognosis for HCC patients are generally determined using the Barcelona Clinical Liver Cancer (BCLC) staging system based on tumor burden, tumor staging, and liver function. Additionally, other prognostic scores such as the ALBI and ART scores have emerged, showing potential relevance in TACE patients [2]. It is recommended that patients with BCLC 0 (very early stage) and BCLC A (early stage) undergo curative treatment involving surgical resection, liver transplantation, and/or local tumor ablation. For patients in the intermediate stage (BCLC B), transarterial chemoembolization (TACE) is recommended as a first-line treatment. There is a growing body of evidence supporting the use of locoregional therapy (LRT), i.e., TACE, radiofrequency ablation, or its combination, to improve clinical outcomes in cases of localized unresectable HCC [3,4]. However, the therapeutic outcomes of LRT are difficult to predict. Disease progression often occurs along the margins of the treated zone due to the development of untreated lesions and/or a failure to treat microsatellite lesions around HCC nodules. The notable recurrence pattern associated with HCC has been described as a double-peaked incidence, referring to early and late recurrences [5]. Early recurrence, which accounts for approximately 40% of HCC patients after LRT [6], generally involves intrahepatic local recurrence, whereas late recurrence generally involves de novo tumor development. Numerous researchers have reported that early recurrence after curative treatment is strongly associated with a poor prognosis. Thus, it is important to identify risk factors for the management of patients who are susceptible to early recurrence via prompt re-intervention. Researchers have identified several risk factors related to intrahepatic recurrence after LRT, including tumor size, tumor number, tumor location, serum tumor markers, and poor differentiation [7,8]. Note, however, that it can be difficult to determine a suitable therapeutic response based on current imaging modalities, such as ultrasound, computed tomography (CT), and magnetic resonance imaging (MRI). Note also that these imaging modalities expose patients to radiation or toxic contrast agents and lack the ability to detect small nodules. In the detection of small HCC tumors, the sensitivity and specificity of these methods can vary considerably: MRI (78.82% and 78.46%), CT (62.35% and 73.85%), and ultrasound (44% and 92%) [9,10]. Furthermore, a recent meta-analysis comparing yttrium-90 radioembolization (Y90RE) and TACE suggests both methods exhibit similar impacts on survival, response rate, and safety, although radioembolization may offer a superior delay in tumor progression [11]. In light of evaluating post-treatment outcomes, recent research suggests that a transient elevation in transaminase levels post-TACE may serve as an indicator of objective response, offering a straightforward tool for a tailored HCC treatment approach [12].

Serum tumor markers are commonly used for HCC surveillance and the evaluation of therapeutic responses. Alpha-fetoprotein (AFP) is a widely used biomarker due to its objectivity, reasonable cost, and convenience; however, the sensitivity and specificity are insufficient for clinical use, and only 30% of patients present an observable increase in AFP serum levels. Researchers have developed other serum tumor markers for clinical use, including AFP-L3%, lectin-bound AFP, and PIVKA-II (protein induced by vitamin K absence or antagonists-II); however, those markers are unable to detect small HCC tumors in roughly 30% of patients [13].

There is a growing body of evidence indicating the potential value of liquid biopsy in precision medicine for the treatment of cancer. Cell-free DNA in liquid biopsies can be used to obtain important genetic and epigenetic information related to malignant cells to facilitate cancer management, including screening, diagnosis, prognosis, and treatment. DNA methylation is an important epigenetic function that plays a fundamental role in every stage of carcinogenesis by regulating tumor-related gene expression. It is believed that DNA hypermethylation is involved in repressing tumor suppressors and DNA repair genes, whereas DNA hypomethylation appears to be responsible for the overexpression of oncogenes in many types of cancer [14,15]. In some cases, genes that undergo aberrant methylation can be used as tumor markers. Examples include cytosine methylation, which involves the covalent binding of a methyl group to genomic DNA. Note that it provides stability superior to that of protein or RNA markers [16]. The detection of methylation markers in liquid biopsy (e.g., blood, urine, saliva, and stool) provides a non-invasive method to monitor cancer progression [17].

A number of studies have reported on the clinical application of methylation markers in cancer diagnosis and prognosis; however, no previous studies have mentioned the application of methylation markers in HCC as a tumor biomarker after LRT. This is the first study to assess the feasibility of using methylation markers from liquid biopsy—blood to predict the response of HCC patients to LRT. We also compared the effectiveness of methylation markers with that of existing tumor markers.

## 2. Materials and Methods

### 2.1. Patients

This study prospectively enrolled 110 treatment-naïve BCLC-B HCC patients who were initially treated with LRT, including TACE with/without radiofrequency ablation (RFA), at National Taiwan University Hospital between June 2020 and May 2021. The diagnosis of HCC and TACE eligibility were assessed prior to treatment using contrast-enhanced CT or MRI in accordance with the guidelines of the American Association for the Study of Liver Disease [18]. At least two dynamic imaging modalities were required for diagnosis. Pathological tissue samples were obtained from one patient with an atypical vascular profile in imaging studies and low AFP levels. Inclusion criteria included the detection of HCCs according to the criteria of BCLC stage B. Exclusion criteria included an inability to tolerate TACE due to renal insufficiency, an allergy to contrast agents, or elevated total bilirubin (TBIL) levels (>2.5 mg/dL). This study was approved by the Research Ethics Committee of the National Taiwan University Hospital (NTUH REC: 202009079DIPB; ClinicalTrials: NCT03267290). All patients provided written informed consent. 

### 2.2. Procedures

#### 2.2.1. TACE Protocol

Following the selection of feeding vessels within HCCs, subsegmental catheterization was performed using microcatheters under abdominal angiography. TACE was performed using an emulsion of 2–10 mL of iodized oil and 10–40 mg of doxorubicin hydrochloride (Adriblastina; Pfizer, Milano, Italy) based on the size of the tumor. Note that the infusion continued until arterial flow stasis was reached. Subsequent embolization of the feeding artery was achieved using Gelfoam cubes (SPONGOSTAN^TM^; Ferrosan Medical Devices, Søborg, Denmark). Technical success was defined as no visible residual tumor stain in post-embolization celiac or common hepatic arteriography.

#### 2.2.2. RFA Protocol

RFA was administered as an add-on procedure three days after TACE in the event that viable tumors were observed after TACE. All patients underwent moderate intravenous conscious sedation and local infiltration of 2% lidocaine throughout the procedure. Percutaneous RFA was performed on all residual tumors under ultrasound guidance using RFA electrodes with a 200-W generator (Cool-tip^TM^; Valleylab, Minneapolis, MN, USA). LRT on all nodules was performed whenever possible. The technical success of RFA was defined as the complete ablation of the HCC with a surrounding safety margin of 0.5–1.0 cm in immediate follow-up CT images. 

### 2.3. Follow-Up

Within one week prior to LRT and 30 days after LRT, the patients were assessed in terms of AFP, PIVKA-II, and methylation marker levels, as well as underwent contrast-enhanced imaging analysis (dynamic CT or dynamic MRI). We also recorded demographic profiles (age and gender) as well as tumor characteristics and biochemistry details (hepatitis B surface antigen, anti-hepatitis C virus antibody, tumor size, tumor number, TBIL, albumin, alanine aminotransferase (ALT), aspartate aminotransferase (AST), creatinine levels (CRE), and platelet count). Patients' liver function and disease status were evaluated using Child-Pugh score classification [19], the ALBI (Albumin-Bilirubin) grade system [20], and Up-to-7 criteria [21] as appropriate. Follow-up imaging was continued at intervals of three months for a period of two years. Tumor recurrence was diagnosed using the same criteria as the American Association for the Study of Liver Disease guidelines [18]. Disease progression was defined as a >20% increase in the sum of the diameters of viable target lesions or newly developed lesions based on modified response evaluation criteria for solid tumors [22].

### 2.4. Cell-Free DNA Extraction from Plasma Samples

Blood samples were collected in Cell-Free DNA BCT^®^ (Streck, La Vista, NE, USA) and further processed to plasma in less than 10 days. The plasma fraction was separated from the blood cells by centrifugation for 15 min at room temperature at 2000× *g*. The collected plasma was aliquoted and stored at −80 °C until use. cfDNA was extracted from plasma by using the QIAamp DNA Blood Mini Kit (Qiagen, Redwood City, CA, USA) according to the manufacturer’s instructions.

### 2.5. Real-Time Quantitative Methylation Analysis

Cell-free DNA from plasma samples was bisulfite-converted using the EZ DNA Methylation Kit (Zymo Research, Irvine, CA, USA) and amplified via real-time quantitative methylation-specific PCR (qMSP) using fluorescent probes. Each reaction was performed using 1x Kapa Probe Fast qPCR Master Mix with 0.5 μM of each primer and 0.25 μM of the probe in a total volume of 20 μL. Amplification was performed on a QuantStudio 5 real-time PCR System (Thermo Fisher Scientific, Waltham, MA, USA) under the following thermocycling conditions: 94 °C for 7 min, followed by 45 cycles of 94 °C for 30 s, 62 °C for 30 s, and 72 °C for 30 s. Methylation levels were calculated as the difference in Ct value between β-actin and candidates using the following formula: 2^[Ct (β-actin) − Ct (candidate)]^ × 1000 in accordance with the protocol outlined in [23]. Primer and probe sequences are available upon request.

### 2.6. Statistical Analysis

A paired-sample *t*-test was used to measure differences in methylation levels before and after treatment for 30 days. Logistic regression models were used to establish two prediction models for therapeutic response and prognosis. Diagnostic effects were assessed using receiver operating characteristic curve analysis to estimate the area under the curve (AUC), cutoff value, sensitivity, and specificity. Univariate COX regression analysis was used to assess the correlation between variables and survival. Survival curves were calculated using the Kaplan-Meier method, and distributions were compared using the log-rank test. Disease-free survival was calculated from the date of diagnosis until recurrence or the end of follow-up, and overall survival was calculated from the date of diagnosis until disease-caused death or the end of follow-up. Cox proportional hazard models were used for multivariate analysis in estimating hazard ratios (HRs) and the corresponding 95% confidence intervals (CIs). Statistical significance was accepted when *p* < 0.05 for all tests.

## 3. Results

### 3.1. Clinicopathological Characteristics of Patients

This study recruited 110 HCC patients with BCLC stage B who underwent LRT. The patients were divided into three groups based on their response to treatment: complete response (N = 53, 48.1%), stable disease or partial response (N = 38, 34.6%), and progressive disease (N = 19, 17.3%). The 72 patients with complete responses and progressive disease were enrolled for outcome analysis with a median follow-up of 2.4 years. The details of patients’ clinicopathological characteristics are listed in Table 1.

#### Correlation between Methylation Markers and Responses of LRT

**Figure 1:**
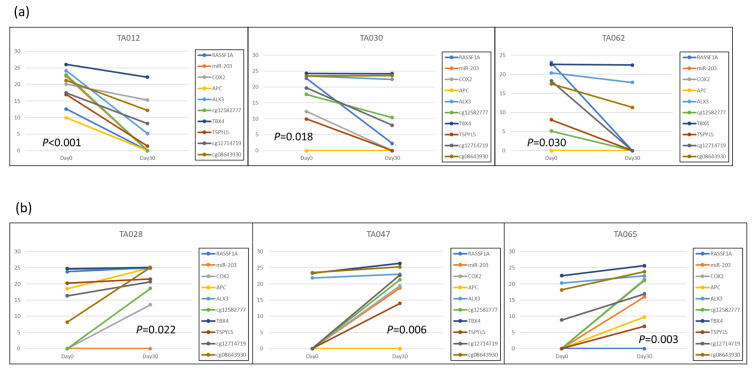
Methylation level changes of candidate biomarkers following LRT: (**a**) The representative three patients (TA012, TA030, TA062) with complete response after LRT; (**b**) The representative three patients (TA028, TA047, TA065) with disease progression after LRT. The *Y*-axis represents methylation levels, and the *X*-axis indicates two-time points, 0 days and 30 days.

There are no reports that have shown that the methylation level will change after LRT. Based on our previous work [24,25], we selected several HCC-specific methylation regions, including Ras association domain family protein 1 isoform A (RASSF1A), MicroRNA 203 (miR-203), cyclooxygenase 2 (COX2), adenomatous polyposis coli (APC), aristaless-like homeobox 3 (ALX3), cg12582777, T-Box transcription factor 4 (TBX4), testis-specific Y-encoded protein-like protein 5 (TSPYL5), cg12714719, and cg08643930, to test their methylation levels before and after LRT. We found that the methylation levels of patients with complete response (CR) or progressive disease (PD) presented distinctly different patterns. In the representative CR patients, the methylation levels of candidate markers tended to decline at 30-day follow-up (Day 30) after LRT compared with before treatment (Day 0). By contrast, PD patients tended to have elevated methylation levels at Day 30 (Figure 1). These results indicate that the change in DNA methylation levels reflects the treatment response and imply that these methylation candidates may act as assessment biomarkers in LRT.

### 3.2. Performance of the Methylation Monitoring Model for LRT Response

**Figure 2:**
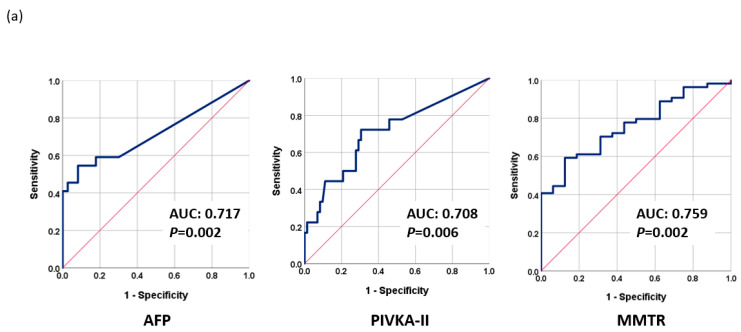

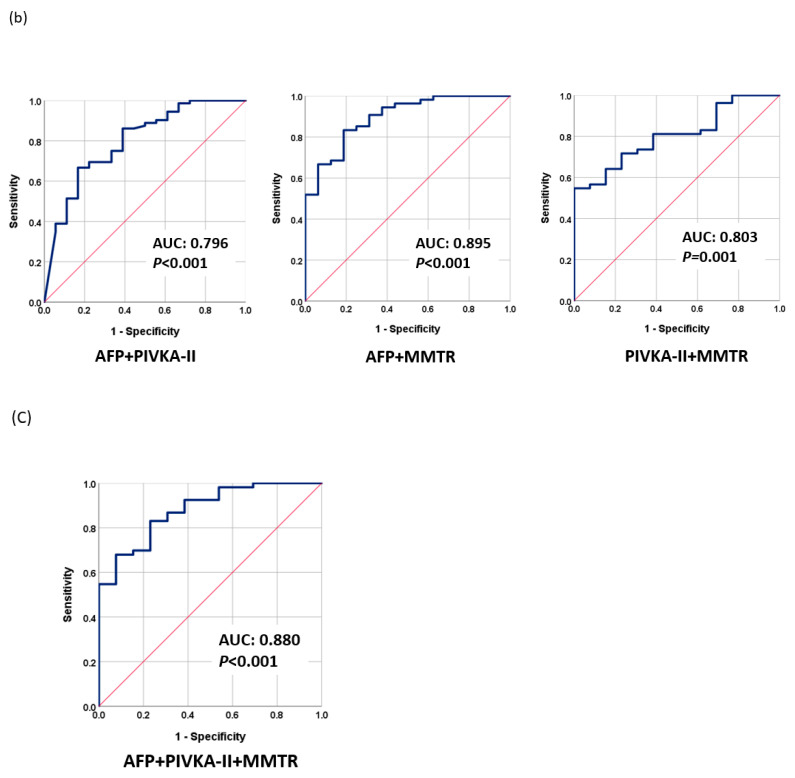
Performance of tumor markers in monitoring therapeutic response as evaluated using receiver operating characteristic analysis (ROC): (**a**) AFP, PIVKA-II, and MMTR; (**b**) AFP + PIVKA-II, AFP + MMTR, and PIVKA-II + MMTR; (**c**) MMTR index + AFP + PIVKA-II.

The proposed methylation monitoring model for tumor response (MMTR) was based on logistic regression analysis and calculated using the blood methylation levels of 10 selected markers, as follows: MMTR index = 1.457 + 0.035 × (methylation level (Δ) of RASSF1A) − 0.002 × (methylation level (Δ) of APC) + 0.068 × (methylation level (Δ) of cg12714719) + 0.038 × (methylation level (Δ) of cg08643930) + 0.017 × (methylation level (Δ) of miR203) − 0.047 × (methylation level (Δ) of COX2) − 0.049 × (methylation level (Δ) of ALX3)-0.069 × (methylation level (Δ) of TSPYL5) + 0.032 × (methylation level (Δ) of cg12582777) + 0.147 × (methylation level (Δ) of TBX4). Note that methylation level (Δ) indicates the change in methylation levels between Day 0 and Day 30. The performance of MMTR in discriminating between CR and PR after LRT was evaluated by receiver operating characteristic (ROC) analysis. The area under the curve (AUC) of the MMTR index was 0.759 (95% CI: 0.641–0.877, *p* = 0.002) (Figure 2a). We examined the performance of AFP and PIVKA-II for the therapeutic response assessment in the same patient group. The AUCs of AFP and PIVKA-II were 0.717 (95% CI: 0.572–0.862, *p* = 0.002) and 0.708 (95% CI: 0.565–0.852, *p* = 0.006), respectively (Figure 2a). Combining the serum markers AFP and PIVKA-II increased the AUC to 0.796 (95% CI: 0.678–0.915, *p* < 0.001). Integrating the MMTR index with AFP or PIVKA-II dramatically increased the AUCs to 0.895 (95% CI: 0.812–0.977, *p* < 0.001) or 0.803 (95% CI: 0.691–0.914, *p* = 0.001), respectively (Figure 2b). Combining the MMTR index, AFP, and PIVKA-II further increased the AUC to 0.880 (95% CI: 0.786–0.973, *p* < 0.001) (Figure 2c).

The MMTR index had a sensitivity of 72.2% and a specificity of 62.5%, while AFP had a sensitivity of 59.1% and a specificity of 82.2%, and PIVKA-II had a sensitivity of 72.2% and a specificity of 69.4%. The various combinations of these biomarkers had different sensitivities and specificities, with the combination of the MMTR index + AFP providing the most effective assessments with a sensitivity of 83.3% and a specificity of 81.2%. Overall, the combination of the MMTR index with AFP was found to be the most effective in assessing LRT response (Table 2a).

#### 3.2.1. Prediction of Early Progression in HCC Patients before LRT

**Figure 3:**
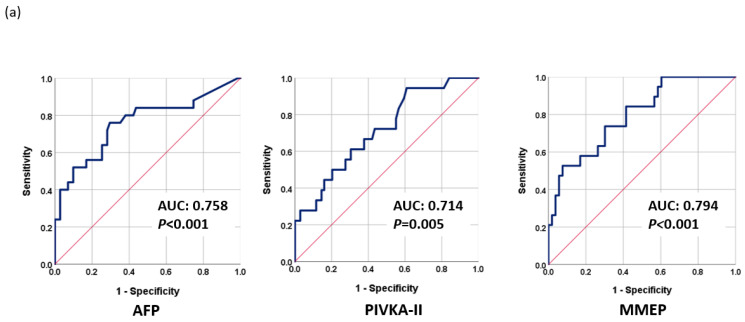

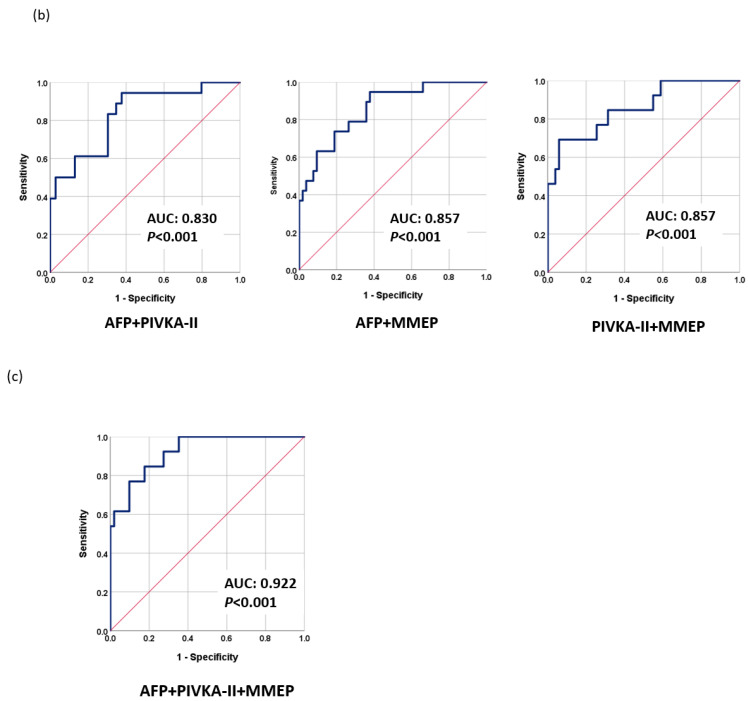
Performance of tumor markers in predicting early progression to late stage as evaluated using receiver operating characteristic analysis (ROC): (**a**) AFP, PIVKA-II, and MMEP; (**b**) AFP + PIVKA-II, AFP + MMTR, and PIVKA-II + MMTR; (**c**) MMTR + AFP + PIVKA-II.

The fact that TACE refractoriness and early progression to late stage are common in intermediate-stage HCC patients and are difficult to predict greatly undermines LRT outcomes. We found that the methylation levels before LRT (Day 0) may reflect the early progression of HCC. Therefore, we used the baseline methylation levels of selected markers to construct a methylation prediction model for early progression (MMEP), as follows: MMEP index = −4.333 − 0.064 × (methylation level (D0) of RASSF1A) − 0.058 × (methylation level (D0) of miR203) + 0.001 × (methylation level (D0) of COX2) + 0.176 × (methylation level (D0) of APC) + 2.121 × (methylation level (D0) of ALX3) − 0.035 × (methylation level (D0) of cg12582777)-0.139 × (methylation level (D0) of TBX4) + 0.01 × (methylation level (D0) of TSPYL5)-0.044 × (methylation level (D0) of cg12714719) + 0.138 × (methylation level (D0) of cg08643930). We then evaluated the performance of the MMEP index in terms of AUC as follows: MMEP (0.794; 95% CI: 0.681–0.908, *p* < 0.001), AFP (0.758; 95% CI: 0.636–0.880, *p* < 0.001), and PIVKA-II (0.714; 95% CI: 0.583–0.844, *p* = 0.005) (Figure 3a). The AUC of the various combinations were as follows: AFP + PIVKA-II (0.830; 95% CI: 0.723–0.937, *p* < 0.001), MMEP + AFP (0.857; 95% CI: 0.762–0.952, *p* < 0.001), and MMEP + PIVKA-II (0.857; 95% CI: 0.736–0.977, *p* < 0.001) (Figure 3b). The best result was obtained for MMEP + PIVKA-II + AFP (0.922; 95% CI: 0.848–0.995, *p* < 0.001) (Figure 3c).

The MMEP index had a sensitivity of 73.7% and a specificity of 69.8%, while AFP had a sensitivity of 76.0% and a specificity of 70.4%, and PIVKA-II had a sensitivity of 66.7% and a specificity of 62.3%. The various combinations of these biomarkers had different sensitivities and specificities, with the combination of MMEP, AFP, and PIVKA-II providing the most effective assessments with a sensitivity of 92.3% and a specificity of 72.5%. Overall, the combination of MMEP with AFP and PIVKA-II was found to be the most effective in predicting the prognosis prior to LRT treatment (Table 2b).

#### 3.2.2. Factors Associated with the Therapeutic Outcomes of LRT

The predictors with a significant effect in the assessment of LRT response and early disease progression were identified by examining several clinicopathological factors, tumor markers, and methylation markers using univariate and multivariate logistic regression analysis. The factors included tumor size, tumor number, TBIL, AST, ALT, PLT, CRE, Child-Pugh score, albumin-bilirubin (ALBI) grade, Up-to-7 criteria, AFP, PIVKA-II, and the proposed methylation indexes. In univariate analysis, significant factors for monitoring LRT response included Up-to-7, AFP, and MMTR index, while in multivariate analysis, AFP and MMTR index were significant (Table 3). In univariate analysis of early tumor progression, significant factors included tumor size, tumor number, PLT count, Up-to-7, and MMEP index, while in multivariate analysis, PLT count, Up-to-7, and MMEP index were significant (Table 4). Overall, the results indicate that methylation markers were more effective than AFP and PIVKA-II in monitoring LRT response and predicting early tumor progression. 

#### 3.2.3. Predicting Survival Using Serum Tumor Markers and MMEP

The potential risk factors for disease-free survival and overall survival were determined using the Cox proportional hazards regression model. In univariate analysis, significant independent risk factors for disease-free survival included tumor size, PLT, ALBI grade, PIVKA-II, and MMEP index, while in multivariate analysis, ALBI grade and MMEP were significant (Table 5). In univariate analysis of overall survival, significant independent risk factors included tumor size, TBIL, AST, PIVKA-II, and the MMEP index, while in multivariate analysis, only the MMEP index was identified as a significant independent risk factor (Table 6). Overall, the results indicate that the MMEP index was a significant independent risk factor for both disease-free survival and overall survival.

**Figure 4:**
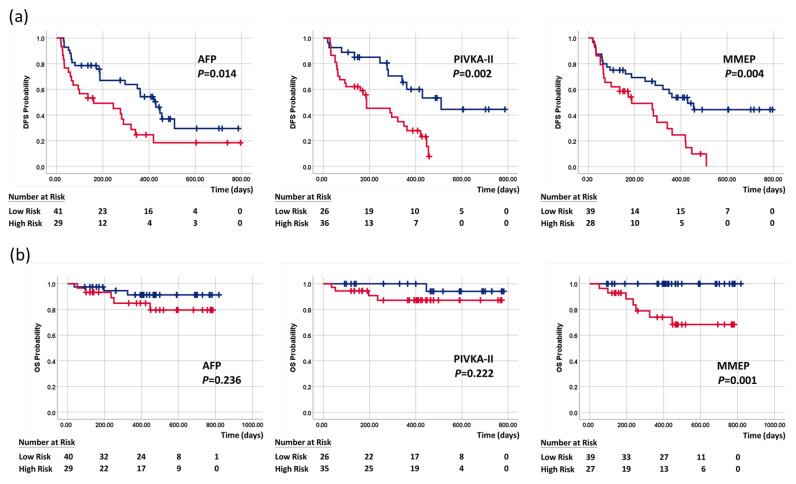
Kaplan–Meier curves of (**a**) disease-free survival and (**b**) overall survival stratified by AFP, PIVKA-II, and MMEP index. Blue line indicates low-risk patients, and red line is high-risk patients.

The serum markers and MMEP index were examined using the Kaplan–Meier method to determine their predictive ability for overall survival and disease-free survival (see Figure 4a,b). The two-year disease-free survival rates of low-profile and high-profile patients were respectively as follows: AFP (29.6% and 18.5%; mean disease-free survival: 14.1 vs. 9.2 months, *p* = 0.014), PIVKA-II (44.4% and 7.7%; mean disease-free survival: 17.0 vs. 7.7 months, *p* = 0.002), and MMEP index (44.3% and 0%; mean DFS: 15.5 and 7.7 months, *p* = 0.004). In our analysis of overall survival, only the MMEP index was significant (mean overall survival: 24 vs. 7 months, *p* = 0.001). These results demonstrated the impressive prognostic ability of these methylation markers, far exceeding current clinicopathological factors and serum markers in assessing disease-free survival and overall survival.

## 4. Discussion

Tumor markers are critical to monitoring the therapeutic effects and early detection of HCC recurrence. Contrast-enhanced imaging has also proven useful; however, it is somewhat limited during post-operative follow-up due to the toxicity of contrast agents and their high costs. AFP is currently the serum marker most widely used for HCC screening, diagnosis, and the evaluation of therapeutic efficacy; however, more than 35% of HCC patients do not present elevated serum AFP levels even in the late stages [26]. One multicenter study reported AFP levels lower than 20 ng/mL in 53.5% of cases involving early HCC and 41.5% of cases involving late HCC [27]. Furthermore, PIVKA-II is no better than AFP in the detection of HCC [28]. In the current study, 41 out of 72 patients (56.9%) were deemed AFP negative under a cut-off value of 20 ng/mL, and 27 out of 65 patients (41.5%) were deemed PIVKA-II negative at a cut-off value of 40 mAU/mL. The fact that these patients presented normal serum tumor markers means that they are ineligible for any assessments other than imaging. By contrast, the methylation indexes in this study are applicable to all patients. 

DNA methylation is an important epigenetic phenomenon that manages gene expression in every phase of carcinogenesis. Numerous studies have reported that in many types of cancer, hypermethylation downregulates the expression of tumor suppressor genes or DNA repair genes, while hypomethylation upregulates various oncogenes [14,15]. This means that the aberrant methylation of genes could potentially be used as an indicator of cancer for diagnosis and prognosis prediction [29]. This is the first study to explore the possibility of using methylation markers to assess the therapeutic response to LRT in HCC patients. The MMTR index based on 10 DNA methylation profiles was shown to vary consistently with the response to LRT among all HCC patients in a clinical setting. Overall, the proposed index performed at least as well as AFP and PIVKA-II in terms of prediction ability. Combining the proposed index with conventional tumor markers resulted in outstanding prediction ability (AUC = 0.880) with good sensitivity (75.0%) and specificity (66.7%).

BCLC guidelines recommend LRT as a standard treatment for patients with intermediate-stage (BCLC B stage); however, the high degree of variability in this group (in terms of tumor burden, biologic behavior, and liver function) can make it very difficult to characterize the therapeutic response to LRT or formulate a reliable prognosis. It is essential that physicians have the ability to identify patients for whom LRT would provide no benefit and those who are at risk of early progression to late-stage cancer, thereby making it possible to initiate alternative systemic therapies [30,31]. Researchers have compiled an extensive list of factors that are correlated with HCC recurrence, including tumor size, satellite tumor, capsular integrity, proximity to large vessels, vascular invasion, partial necrosis, AFP level, platelet count, antiviral treatment, and viral etiology [8,32]. In the current study, we sought to identify biomarkers associated with DNA methylation with which to formulate prognostic predictions for LRT.

This was achieved by constructing a methylation assessment model (MMTR) for LRT and a methylation prognostic model before therapy (MMEP) based on 10 methylation regions RASSF1A, miR-203, COX2, APC, ALX3, TBX4, TSPYL5, cg12582777, cg12714719, and cg08643930. In a previous study, we demonstrated the correlation between HCC and RASSF1A, miR-203, APC, and COX2 [25]. All three of these genes and one microRNA present the characteristics of tumor suppressors and have an outstanding ability to identify HCC at an early stage. It is also possible that these genes could be used as prognostic markers by which to predict the survival of HCC patients with a high degree of specificity. ALX3 is a member of the homeobox family with oncogenic potential in cervical squamous cell carcinoma through the transactivation of CDC25A by recruiting lysine demethylase 2B [33]. The methylation of ALX3 has been associated with neuroblastoma, colorectal carcinoma, and HCC [34,35]. TBX4 down-regulation is an independent prognostic marker for survival in patients with stage II pancreatic ductal adenocarcinoma [36]. TBX4 hypermethylation has been observed in bladder cancer and lung cancer [37,38]. TSPYL5 repression via DNA methylation is frequently associated with cancer, including endometrioid endometrial adenocarcinoma, malignant glioma, gastric cancer, prostate cancer, and HCC [39,40,41,42,43]. The expression of TSPYL5 can be used as a prognostic indicator for gastric cancer and breast cancer [44]. This work extends the clinical application of these methylation markers to HCC.

AFP is the most widely used marker for HCC surveillance due to its close association with tumor differentiation, vascular invasion, and progression of HCC tumors. Overall, we determined that AFP was more effective than PIVKA-II in predicting the response to LRT; however, the proposed MMTR index (AUC = 0.759, *p* = 0.002) outperformed both AFP (AUC = 0.717, *p* = 0.002) and PIVKA-II (AUC = 0.708, *p* = 0.006). As shown in Figure 2, the high AUC values obtained by combining MMTR + AFP (0.895), MMTR + PIVKA-II (0.803), and MMTR + AFP + PIVKA-II (0.880) indicate that the overall most effective approach to predicting the treatment response involved combining the analysis results of AFP and methylation markers.

Our clinical data revealed that 26.4% of patients rapidly progressed to the late stage soon after LRT. It is possible that this can be attributed to the limitations of imaging tools and the shortcomings of current serum markers in formulating pre-operative evaluations. In predicting the prognosis prior to treatment, methylation markers proved superior to AFP or PIVKA-II (Figure 3). Combining MMEP + AFP or PIVKA-II resulted in similar prediction performance (AUC = 0.857); however, combining MMEP + AFP + PIVKA-II resulted in a very high AUC of 0.922 (*p* < 0.001). These results may reflect the biological characteristics of methylation markers, whose levels change very early (i.e., before the appearance of symptoms). The integration of both information from image tools and effective biomarkers provides a precise assessment tool for surveillance and prognosis and could help physicians make a more effective treatment strategy, including an earlier application of adjuvant therapy and more frequent monitoring.

In acknowledging the limitations of our study, it is important to note that the sample size is indeed limited, which could potentially impact the generalizability of our findings. This is a significant constraint that future research should seek to address by including a more extensive patient cohort. Additionally, our study did not incorporate an external validation cohort, which might have strengthened the reliability and applicability of our results. Future studies would benefit from incorporating such a validation set to bolster the robustness of the findings.

## 5. Conclusions

This is the first study to prove the clinical utility of methylation markers for the assessment of LRT response and prognosis, with performance superior to that of current serum markers, including AFP and PIVKA-II. The promising results indicate that methylation markers potentially provide a useful tool for managing HCC patients who are at high risk for close surveillance or adjuvant treatment after LRT. It may even be possible to predict tumor progression with a high degree of precision prior to treatment, thereby making it possible to formulate an effective therapeutic strategy to improve HCC outcomes.

## Figures and Tables

**Table 1:** Clinicopathological characteristics of patients.

Gender	
Male (%)	53 (73.6)
Female (%)	19 (26.4)
Age, median (range)	68.5 (45–94)
Hepatitis Status	
HBV (%)	35 (48.6)
HCV (%)	21 (29.2)
HBV(+)/HCV(+) (%)	1 (1.4)
HBV(−)/HCV(−) (%)	15 (20.8)
Tumor Size, median (range)	2 (0.7–16)
Tumor Number	
1 (%)	48 (66.7)
2 (%)	9 (12.5)
≥3 (%)	15 (20.8)
AFP	
<20 ng/mL (%)	42 (58.3)
PIVKA-II	
<40 mAU/mL (%)	27 (41.2)
ALBI Grade	
1 (%)	54 (75)
2 (%)	18 (25)
Child-Pugh Score	
A (%)	66 (91.7)
B (%)	6 (8.3)
Up-to-7 Criteria	
In (%)	53 (73.6)
Out (%)	19 (26.4)
Response	
CR (%)	53 (73.6)
PD (%)	19 (26.4)
Tumor Progression within one year	
Early progression (+) (%)	52 (73.6)
Early progression (−) (%)	20 (26.4)

HBV, hepatitis B virus; HCV, hepatitis C virus; AFP, alpha-fetoprotein; PIVKA-II, Protein Induced by Vitamin K Absence or Antagonist-II; ALBI, albumin-bilirubin; CR, complete response; PD, progressive disease.

**Table 2:** Sensitivity and specificity of current tumor markers and methylation prediction models for treatment response and early progression. (a) Assessment of treatment response. (b) Prediction of early progression before treatment.

(a)				
	AUC (95% CI)	Sensitivity (%)	Specificity (%)	*p*-Value
AFP	0.717 (0.572–0.862)	59.1	82.2	0.002
PIVKA-II	0.708 (0.565–0.852)	72.2	69.4	0.006
MMTR index	0.759 (0.641–0.877)	72.2	62.5	0.002
AFP + PIVKA-II	0.796 (0.678–0.915)	75.0	66.7	<0.001
MMTR + AFP	0.895 (0.812–0.977)	83.3	81.2	<0.001
MMTR + PIVKA-II	0.803 (0.691–0.914)	71.7	76.9	0.001
MMTR + AFP + PIVKA-II	0.880 (0.786–0.973)	83.0	76.9	<0.001
**(b)**				
	**AUC (95% CI)**	**Sensitivity (%)**	**Specificity (%)**	***p*-Value**
AFP	0.758 (0.636–0.880)	76.0	70.4	<0.001
PIVKA-II	0.714 (0.583–0.844)	66.7	62.3	0.005
MMEP index	0.794 (0.681–0.908)	73.7	69.8	<0.001
AFP + PIVKA-II	0.830 (0.723–0.937)	83.3	69.6	<0.001
MMEP + AFP	0.857 (0.762–0.952)	78.9	73.6	<0.001
MMEP +PIVKA-II	0.857 (0.736–0.977)	84.6	68.6	<0.001
MMEP +AFP + PIVKA-II	0.922 (0.848–0.995)	92.3	72.5	<0.001

AFP, alpha-fetoprotein; PIVKA-II, Protein Induced by Vitamin K Absence or Antagonist-II; MMTR, Methylation prediction model for tumor response; MMEP, methylation prediction model for early progression; AUC, area under the curve.

**Table 3:** Univariate and multivariate analysis of predictors for LRT response.

	Univariate Analysis			Multivariate Analysis		
	HR	95% CI	*p* Value	HR	95% CI	*p* Value
Tumor size	0.855	0.709–1.031	0.101	-	-	-
Tumor number	0.684	0.387–1.208	0.190	-	-	-
Albumin	1.005	0.596–1.696	0.984	-	-	-
TBIL	0.743	0.494–1.116	0.152	-	-	-
AST	0.996	0.986–1.005	0.362	-	-	-
ALT	0.994	0.982–1.006	0.303	-	-	-
PLT count	0.992	0.983–1.001	0.067	-	-	-
CRE	0.398	0.026–6.137	0.509	-	-	-
Child-Pugh score	1.600	0.174–14.73	0.678			
ALBI grade	0.512	0.157–1.670	0.267			
Up-to-7	0.281	0.088–0.896	0.032	1.648	0.268–10.12	0.590
AFP	0.178	0.059–0.537	0.002	0.104	0.024–0.443	0.002
PIVKA-II	0.533	0.264–1.076	0.079	-	-	-
MMTR index	209.95	5.125–8600.35	0.005	1452.15	9.18–229,712.9	0.005

HR, hazard ratio; CI, confidence interval; TBIL, total bilirubin; ALT, alanine aminotransferase; AST, aspartate aminotransferase; PLT, platelet; CRE, creatinine; ALBI, albumin-bilirubin; AFP, alpha-fetoprotein; PIVKA-II, Protein Induced by Vitamin K Absence or Antagonist-II; MMTR, Methylation prediction model for tumor response.

**Table 4:** Univariate and multivariate analysis of predictors for early progression.

	Univariate Analysis			Multivariate Analysis		
	HR	95% CI	*p* Value	HR	95% CI	*p* Value
Tumor size	1.320	1.051–1.656	0.017	0.879	0.544–1.418	0.596
Tumor number	1.746	0.999–3.052	0.051	1.191	0.457–3.103	0.721
Albumin	0.551	0.130–2.331	0.418	-	-	-
TBIL	1.382	0.676–2.827	0.375	-	-	-
AST	1.007	0.996–1.018	0.197	-	-	-
ALT	1.005	0.989–1.021	0.570	-	-	-
PLT	1.008	1.001–1.015	0.032	1.011	1.000–1.022	0.044
CRE	0.586	0.081–2.230	0.596	-	-	-
Child-Pugh score	1.237	0.209–7.327	0.815			
ALBI grade	2.523	0.824–7.730	0.105			
Up-to-7	8.381	2.586–27.17	0.000	10.369	1.205–89.23	0.033
AFP	1.004	1.000–1.007	0.062	-	-	-
PIVKA-II	1.001	1.000–1.002	0.209	-	-	-
MMEP index	175.06	8.876–33,452.5	0.001	240.76	4.888–11,859.0	0.006

HR, hazard ratio; CI, confidence interval; TBIL, total bilirubin; ALT, alanine aminotransferase; AST, aspartate aminotransferase; PLT, platelet; CRE, creatinine; ALBI, albumin-bilirubin; AFP, alpha-fetoprotein; PIVKA-II, Protein Induced by Vitamin K Absence or Antagonist-II; MMEP, methylation prediction model for early progression.

**Table 5:** Univariate and multivariate analysis of risk factors for disease-free survival.

	Univariate Analysis			Multivariate Analysis		
	HR	95% CI	*p* Value	HR	95% CI	*p* Value
Tumor size	1.161	1.030–1.310	0.015	0.991	0.833–1.179	0.920
Tumor number	1.185	0.855–1.641	0.309	-	-	-
Albumin	0.554	0.227–1.349	0.193	-	-	-
TBIL	1.323	0.881–1.987	0.177	-	-	-
AST	1.003	0.998–1.009	0.256	-	-	-
ALT	1.007	0.998–1.016	0.137	-	-	-
PLT count	1.004	1.000–1.009	0.041	1.004	0.999–1.009	0.125
CRE	1.371	0.418–4.502	0.603	-	-	-
Child-Pugh score	1.508	0.537–4.235	0.435	-	-	-
ALBI grade	1.923	1.026–3.603	0.041	2.671	1.281–5.566	0.009
AFP	1.000	1.000–1.000	0.081	-	-	-
PIVKA-II	1.000	1.000–1.000	<0.001	1.000	1.000–1.000	0.258
MMEP	14.101	3.691–53.87	<0.001	7.423	1.263–43.61	0.027

HR, hazard ratio; CI, confidence interval; TBIL, total bilirubin; ALT, alanine aminotransferase; AST, aspartate aminotransferase; PLT, platelet; CRE, creatinine; ALBI, albumin-bilirubin; AFP, alpha-fetoprotein; PIVKA-II, Protein Induced by Vitamin K Absence or Antagonist-II; MMEP, methylation prediction model for early progression.

**Table 6:** Univariate and multivariate analysis of risk factors for overall survival.

	Univariate Analysis			Multivariate Analysis		
	HR	95% CI	*p* Value	HR	95% CI	*p* Value
Tumor size	1.185	1.018–1.380	0.028	0.722	0.155–3.371	0.678
Tumor number	1.830	0.979–3.420	0.058	-	-	-
Albumin	0.422	0.059–3.005	0.389	-	-	-
TBIL	1.959	1.030–3.728	0.041	1.401	0.294–6.686	0.672
AST	1.011	1.002–1.019	0.017	0.980	0.903–1.064	0.631
ALT	1.006	0.986–1.026	0.584	-	-	-
PLT	1.004	0.996–1.012	0.305	-	-	-
CRE	0.082	0.003–2.027	0.127	-	-	-
Child-Pugh score	4.521	0.910–22.47	0.065	-	-	-
ALBI grade	3.10	0.779–12.50	0.108			
AFP	1.000	1.000–1.000	0.861	-	-	-
PIVKA-II	1.000	1.000–1.000	0.008	1.000	0.998–1.002	0.847
MMEP	37.683	2.897–490.0	0.006	267.609	1.73–41,526	0.030

HR, hazard ratio; CI, confidence interval; TBIL, total bilirubin; ALT, alanine aminotransferase; AST, aspartate aminotransferase; PLT, platelet; CRE, creatinine; ALBI, albumin-bilirubin; AFP, alpha-fetoprotein; PIVKA-II, Protein Induced by Vitamin K Absence or Antagonist-II; MMEP, methylation prediction model for early progression.

## Data Availability

All data generated or analyzed during this study are included in this article. Further inquiries can be directed to the corresponding author.
